# Bis(1,2,2,6,6-penta­methyl­piperidin-4-yl) 2-butyl-2-(3,5-di-*tert*-butyl-4-hydroxy­benz­yl)malonate (Tinuvin 144)

**DOI:** 10.1107/S1600536809035260

**Published:** 2009-09-05

**Authors:** Tao Zeng, Wan-Zhong Ren

**Affiliations:** a Chemistry & Biology College, Yantai University, Yantai 264005, People’s Republic of China

## Abstract

The title compound, C_42_H_72_N_2_O_5_, a hindered amine light stabiliser (HALS) with the trade name Tinuvin 144 was prepared from bis­(1,2,2,6,6-penta­methyl­piperidin-4-yl) 2-butyl­malonate and 2,6-di-*tert*-butyl-4-[(dimethyl­amino)meth­yl]phenol using lithium amide as a catalyst. In the mol­ecule, both piperidine rings adopt chair conformations. In the crystal, inversion dimers linked by pairs of O—H⋯O hydrogen bonds occur.

## Related literature

For further information on Tinuvin 144, see: Eggensperger *et al.* (1974 ▶, 1976 ▶). For background to hindered amine light stabilisers, see: Denisov (1991 ▶); Klemchuk & Gande (1998 ▶); Yamazaki & Seguchi (1997 ▶); Rasberger (1980 ▶). For a related structure, see: Zeng & Chen (2006 ▶).
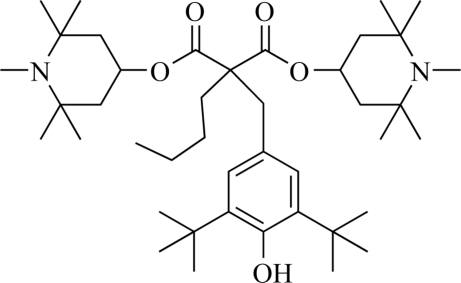


## Experimental

### 

#### Crystal data

C_42_H_72_N_2_O_5_
*M* *_r_* = 685.02
Monoclinic, 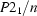

*a* = 13.736 (6) Å
*b* = 18.827 (8) Å
*c* = 17.185 (7) Å
β = 108.679 (8)°
*V* = 4210 (3) Å^3^
*Z* = 4
Mo *K*α radiation
μ = 0.07 mm^−1^
*T* = 294 K
0.24 × 0.22 × 0.20 mm

#### Data collection

Bruker SMART CCD diffractometer
Absorption correction: multi-scan (*SADABS*; Bruker, 1997 ▶) *T*_min_ = 0.984, *T*_max_ = 0.986
23750 measured reflections
8524 independent reflections
4124 reflections with *I* > 2σ(*I*)
*R*_int_ = 0.060

#### Refinement

*R*[*F* ^2^ > 2σ(*F* ^2^)] = 0.051
*wR*(*F* ^2^) = 0.133
*S* = 1.04
8524 reflections
460 parameters
24 restraints
H-atom parameters constrained
Δρ_max_ = 0.15 e Å^−3^
Δρ_min_ = −0.18 e Å^−3^

### 

Data collection: *SMART* (Bruker, 1997 ▶); cell refinement: *SAINT* (Bruker, 1997 ▶); data reduction: *SAINT*; program(s) used to solve structure: *SHELXS97* (Sheldrick, 2008 ▶); program(s) used to refine structure: *SHELXL97* (Sheldrick, 2008 ▶); molecular graphics: *SHELXTL* (Sheldrick, 2008 ▶); software used to prepare material for publication: *SHELXTL*.

## Supplementary Material

Crystal structure: contains datablocks I, global. DOI: 10.1107/S1600536809035260/hb5067sup1.cif

Structure factors: contains datablocks I. DOI: 10.1107/S1600536809035260/hb5067Isup2.hkl

Additional supplementary materials:  crystallographic information; 3D view; checkCIF report

## Figures and Tables

**Table 1:** Hydrogen-bond geometry (Å, °)

*D*—H⋯*A*	*D*—H	H⋯*A*	*D*⋯*A*	*D*—H⋯*A*
O5—H5⋯O4^i^	0.82	2.58	3.200 (3)	134

Symmetry code: (i) 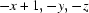.

**Fig. 1.**
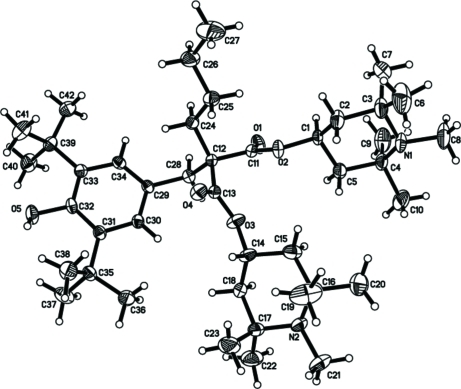
A view of the molecular structure of (I). Displacement ellipsoids are drawn at the 30% probability level and H atoms are shown as small spheres of arbitrary radii.

## supplementary crystallographic information

### Comment

Hindered phenols are widely used as antioxidants while hindered amines are used as light stabilizers in polymers and lubricants both because of their special hindered structures (Denisov, 1991; Klemchuk & Gande ,1998; Yamazaki, 1997). The title compound,*C*_42_H_72_N_2_O_5_, (1), usally called 'Tinuvin144', is a famous light stabilizer of the hinderedamine class that also contains an oxidant unit of the sterically hindered phenol type (Rasberger,1980). In a former paper,we reported bis(1,2,2,6,6-pentamethylpiperidin-4-yl)butylmalonate, a key intermediate in the peparation of Tinuvin 144 (Zeng, 2006). Then Tinuvin 144 was obtained from reaction of bis(1,2,2,6,6-pentamethylpiperidin-4-yl)butylmalonate and 2,6-di-*tert*-butyl-4-((dimethylamino)methyl)phenol catalysized by lithium amide.

In the crystal structure both of the piperidine rings was found to adopt chair confirmations. And the phenolic hydroxyl groups are sterically hindered by the adjacent *tert*-butyl groups.

### Experimental

A mixture of bis(1,2,2,6,6-pentamethylpiperidin-4-yl) 2-butylmalonate (11.67 g,0.025 mol) and 2,6-di-*tert*-butyl-4-((dimethylamino)methyl)phenol (6.59 g, 0.025 mol)was dissloved in toluene (100 ml), stirred and heated to reflux. Then 0.2 g lithium amide was added and stirred for a further 4 h and extracted with water (30 ml) and then dried. The solvent was removed by vacuum evaporation at 318 K, and the product was filtered and washed with methanol (10 ml). Tinuvin 144 (15.05 g) was obtained in 87.9% yield. Colourless blocks of (I) (m.p. 420–422 K) were obtained by slow evaporation of a mixture of THF and methanol.

### Refinement

The O-bound H atom was initially located in a difference map and refined with a distant restraint of 0.82 (1) Å. All H other atoms were positioned geometrically and refined using a riding model, in the range of 0.93–0.98 Å, with *U*_iso_(H) = 1.2*U*_eq_(C).

### Crystal data

**Table:**

C_42_H_72_N_2_O_5_	*F*(000) = 1512
*M**_r_* = 685.02	*D*_x_ = 1.081 Mg m^−^^3^
Monoclinic, *P*2_1_/*n*	Mo *K*α radiation, λ = 0.71073 Å
Hall symbol: -P 2yn	Cell parameters from 4000 reflections
*a* = 13.736 (6) Å	θ = 2.2–21.2°
*b* = 18.827 (8) Å	µ = 0.07 mm^−^^1^
*c* = 17.185 (7) Å	*T* = 294 K
β = 108.679 (8)°	Block, colourless
*V* = 4210 (3) Å^3^	0.24 × 0.22 × 0.20 mm
*Z* = 4	

### Data collection

**Table:**

Bruker SMART CCD diffractometer	8524 independent reflections
Radiation source: fine-focus sealed tube	4124 reflections with *I* > 2σ(*I*)
graphite	*R*_int_ = 0.060
ω scans	θ_max_ = 26.4°, θ_min_ = 1.7°
Absorption correction: multi-scan (*SADABS*; Bruker, 1997)	*h* = −17→12
*T*_min_ = 0.984, *T*_max_ = 0.986	*k* = −23→23
23750 measured reflections	*l* = −11→21

### Refinement

**Table:**

Refinement on *F*^2^	Primary atom site location: structure-invariant direct methods
Least-squares matrix: full	Secondary atom site location: difference Fourier map
*R*[*F*^2^ > 2σ(*F*^2^)] = 0.051	Hydrogen site location: inferred from neighbouring sites
*wR*(*F*^2^) = 0.133	H-atom parameters constrained
*S* = 1.04	*w* = 1/[σ^2^(*F*_o_^2^) + (0.0519*P*)^2^] where *P* = (*F*_o_^2^ + 2*F*_c_^2^)/3
8524 reflections	(Δ/σ)_max_ = 0.002
460 parameters	Δρ_max_ = 0.15 e Å^−^^3^
24 restraints	Δρ_min_ = −0.18 e Å^−^^3^

### Special details

**Table:**

Geometry. All e.s.d.'s (except the e.s.d. in the dihedral angle between two l.s. planes) are estimated using the full covariance matrix. The cell e.s.d.'s are taken into account individually in the estimation of e.s.d.'s in distances, angles and torsion angles; correlations between e.s.d.'s in cell parameters are only used when they are defined by crystal symmetry. An approximate (isotropic) treatment of cell e.s.d.'s is used for estimating e.s.d.'s involving l.s. planes.
Refinement. Refinement of *F*^2^ against ALL reflections. The weighted *R*-factor *wR* and goodness of fit *S* are based on *F*^2^, conventional *R*-factors *R* are based on *F*, with *F* set to zero for negative *F*^2^. The threshold expression of *F*^2^ > σ(*F*^2^) is used only for calculating *R*-factors(gt) *etc*. and is not relevant to the choice of reflections for refinement. *R*-factors based on *F*^2^ are statistically about twice as large as those based on *F*, and *R*- factors based on ALL data will be even larger.

### Fractional atomic coordinates and isotropic or equivalent isotropic displacement parameters (Å^2^)

**Table:**

	*x*	*y*	*z*	*U*_iso_*/*U*_eq_	
O1	0.11801 (11)	0.03727 (8)	0.22237 (9)	0.0545 (4)	
O2	0.27568 (10)	0.03307 (7)	0.31285 (8)	0.0450 (4)	
O3	0.34336 (10)	0.12854 (7)	0.21144 (10)	0.0518 (4)	
O4	0.44526 (10)	0.03948 (7)	0.20033 (9)	0.0472 (4)	
O5	0.34155 (11)	0.00858 (9)	−0.17848 (9)	0.0580 (5)	
H5	0.4033	0.0170	−0.1647	0.087*	
N1	0.26679 (14)	0.08817 (10)	0.54521 (10)	0.0545 (5)	
N2	0.47991 (13)	0.31688 (9)	0.31576 (11)	0.0480 (5)	
C1	0.23602 (16)	0.03915 (11)	0.38210 (12)	0.0400 (5)	
H1	0.1712	0.0131	0.3705	0.048*	
C2	0.31606 (18)	0.00701 (13)	0.45378 (13)	0.0592 (7)	
H2A	0.3812	0.0304	0.4607	0.071*	
H2B	0.3239	−0.0427	0.4423	0.071*	
C3	0.2906 (2)	0.01285 (14)	0.53422 (14)	0.0628 (7)	
C4	0.19126 (18)	0.12587 (12)	0.47700 (14)	0.0505 (6)	
C5	0.22108 (18)	0.11474 (11)	0.39962 (13)	0.0502 (6)	
H5A	0.1678	0.1348	0.3531	0.060*	
H5B	0.2842	0.1405	0.4054	0.060*	
C6	0.3888 (3)	−0.00737 (19)	0.60225 (18)	0.1186 (13)	
H6A	0.4380	0.0304	0.6101	0.178*	
H6B	0.4167	−0.0500	0.5871	0.178*	
H6C	0.3737	−0.0153	0.6523	0.178*	
C7	0.2054 (3)	−0.04052 (15)	0.53448 (18)	0.1022 (11)	
H7A	0.1872	−0.0342	0.5835	0.153*	
H7B	0.2300	−0.0881	0.5329	0.153*	
H7C	0.1461	−0.0325	0.4873	0.153*	
C8	0.2519 (2)	0.10123 (17)	0.62494 (15)	0.0968 (10)	
H8A	0.2521	0.1515	0.6346	0.145*	
H8B	0.3065	0.0793	0.6677	0.145*	
H8C	0.1873	0.0815	0.6245	0.145*	
C9	0.07875 (18)	0.10554 (16)	0.46096 (17)	0.0844 (9)	
H9A	0.0670	0.0585	0.4383	0.127*	
H9B	0.0352	0.1385	0.4228	0.127*	
H9C	0.0634	0.1068	0.5116	0.127*	
C10	0.2038 (3)	0.20520 (14)	0.49609 (18)	0.0944 (10)	
H10A	0.1755	0.2163	0.5390	0.142*	
H10B	0.1682	0.2318	0.4477	0.142*	
H10C	0.2754	0.2173	0.5136	0.142*	
C11	0.20853 (16)	0.03224 (10)	0.23713 (13)	0.0349 (5)	
C12	0.26231 (14)	0.01955 (10)	0.17326 (12)	0.0310 (5)	
C13	0.36247 (16)	0.06151 (11)	0.19717 (12)	0.0340 (5)	
C14	0.42714 (15)	0.17905 (11)	0.24358 (14)	0.0462 (6)	
H14	0.4920	0.1604	0.2397	0.055*	
C15	0.43448 (17)	0.19407 (12)	0.33117 (14)	0.0567 (7)	
H15A	0.4581	0.1515	0.3634	0.068*	
H15B	0.3663	0.2048	0.3332	0.068*	
C16	0.50644 (18)	0.25553 (13)	0.37089 (16)	0.0604 (7)	
C17	0.46967 (17)	0.30720 (11)	0.22855 (15)	0.0481 (6)	
C18	0.39650 (16)	0.24513 (11)	0.19512 (13)	0.0462 (6)	
H18A	0.3281	0.2585	0.1945	0.055*	
H18B	0.3935	0.2359	0.1389	0.055*	
C19	0.61923 (19)	0.23145 (15)	0.3955 (2)	0.1046 (12)	
H19A	0.6321	0.2100	0.3491	0.157*	
H19B	0.6325	0.1975	0.4394	0.157*	
H19C	0.6635	0.2718	0.4133	0.157*	
C20	0.4818 (3)	0.27382 (17)	0.45014 (17)	0.1064 (11)	
H20A	0.5310	0.3076	0.4817	0.160*	
H20B	0.4850	0.2314	0.4819	0.160*	
H20C	0.4140	0.2938	0.4360	0.160*	
C21	0.53831 (19)	0.38095 (12)	0.35218 (17)	0.0761 (8)	
H21A	0.6069	0.3774	0.3497	0.114*	
H21B	0.5407	0.3851	0.4085	0.114*	
H21C	0.5053	0.4221	0.3222	0.114*	
C22	0.4174 (2)	0.37260 (13)	0.18116 (17)	0.0826 (9)	
H22A	0.3587	0.3847	0.1972	0.124*	
H22B	0.3957	0.3627	0.1233	0.124*	
H22C	0.4649	0.4116	0.1931	0.124*	
C23	0.5712 (2)	0.29581 (15)	0.20974 (19)	0.0859 (9)	
H23A	0.6182	0.3335	0.2340	0.129*	
H23B	0.5577	0.2957	0.1514	0.129*	
H23C	0.6009	0.2511	0.2322	0.129*	
C24	0.27941 (14)	−0.06058 (10)	0.16759 (12)	0.0378 (5)	
H24A	0.2125	−0.0832	0.1496	0.045*	
H24B	0.3102	−0.0680	0.1247	0.045*	
C25	0.34472 (17)	−0.09926 (11)	0.24357 (14)	0.0513 (6)	
H25A	0.4141	−0.0804	0.2597	0.062*	
H25B	0.3174	−0.0905	0.2882	0.062*	
C26	0.34825 (19)	−0.17811 (13)	0.23035 (16)	0.0664 (7)	
H26A	0.3787	−0.1868	0.1875	0.080*	
H26B	0.2786	−0.1964	0.2114	0.080*	
C27	0.4086 (2)	−0.21805 (15)	0.3066 (2)	0.1104 (12)	
H27A	0.4786	−0.2018	0.3243	0.166*	
H27B	0.4068	−0.2679	0.2947	0.166*	
H27C	0.3789	−0.2098	0.3493	0.166*	
C28	0.19051 (14)	0.04724 (11)	0.08956 (11)	0.0366 (5)	
H28A	0.1250	0.0230	0.0770	0.044*	
H28B	0.1781	0.0974	0.0950	0.044*	
C29	0.23091 (14)	0.03749 (11)	0.01788 (12)	0.0330 (5)	
C30	0.30080 (14)	0.08459 (11)	0.00438 (12)	0.0357 (5)	
H30	0.3225	0.1227	0.0402	0.043*	
C31	0.34042 (14)	0.07772 (11)	−0.06046 (12)	0.0362 (5)	
C32	0.30647 (15)	0.01948 (11)	−0.11208 (12)	0.0378 (5)	
C33	0.23369 (14)	−0.02878 (10)	−0.10275 (12)	0.0350 (5)	
C34	0.19819 (14)	−0.01768 (11)	−0.03675 (12)	0.0367 (5)	
H34	0.1498	−0.0491	−0.0290	0.044*	
C35	0.41828 (15)	0.13169 (11)	−0.07214 (13)	0.0416 (5)	
C36	0.43759 (18)	0.19175 (13)	−0.00943 (15)	0.0628 (7)	
H36A	0.3741	0.2158	−0.0149	0.094*	
H36B	0.4859	0.2248	−0.0189	0.094*	
H36C	0.4649	0.1725	0.0450	0.094*	
C37	0.37945 (19)	0.16647 (14)	−0.15711 (15)	0.0730 (8)	
H37A	0.3720	0.1309	−0.1987	0.109*	
H37B	0.4279	0.2017	−0.1616	0.109*	
H37C	0.3141	0.1885	−0.1643	0.109*	
C38	0.52270 (16)	0.09604 (13)	−0.05867 (16)	0.0638 (7)	
H38A	0.5436	0.0720	−0.0067	0.096*	
H38B	0.5728	0.1315	−0.0591	0.096*	
H38C	0.5171	0.0623	−0.1018	0.096*	
C39	0.19313 (15)	−0.09002 (11)	−0.16316 (13)	0.0431 (5)	
C40	0.13682 (18)	−0.06090 (14)	−0.24902 (14)	0.0654 (7)	
H40A	0.0782	−0.0338	−0.2475	0.098*	
H40B	0.1145	−0.0997	−0.2868	0.098*	
H40C	0.1824	−0.0309	−0.2665	0.098*	
C41	0.28074 (18)	−0.13933 (13)	−0.16708 (17)	0.0712 (8)	
H41A	0.3264	−0.1137	−0.1891	0.107*	
H41B	0.2525	−0.1793	−0.2017	0.107*	
H41C	0.3180	−0.1558	−0.1128	0.107*	
C42	0.11634 (18)	−0.13606 (13)	−0.13825 (15)	0.0646 (7)	
H42A	0.1493	−0.1560	−0.0849	0.097*	
H42B	0.0924	−0.1737	−0.1775	0.097*	
H42C	0.0591	−0.1074	−0.1368	0.097*	

### Atomic displacement parameters (Å^2^)

**Table:**

	*U*^11^	*U*^22^	*U*^33^	*U*^12^	*U*^13^	*U*^23^
O1	0.0375 (9)	0.0868 (12)	0.0430 (9)	0.0038 (8)	0.0183 (8)	0.0003 (9)
O2	0.0438 (8)	0.0635 (10)	0.0301 (8)	0.0031 (7)	0.0151 (7)	−0.0087 (8)
O3	0.0393 (8)	0.0349 (9)	0.0828 (12)	−0.0058 (7)	0.0218 (8)	−0.0141 (8)
O4	0.0355 (8)	0.0464 (9)	0.0604 (10)	0.0016 (7)	0.0164 (8)	−0.0080 (8)
O5	0.0559 (9)	0.0840 (12)	0.0400 (9)	−0.0131 (9)	0.0236 (8)	−0.0162 (9)
N1	0.0652 (12)	0.0677 (14)	0.0288 (11)	0.0087 (11)	0.0125 (10)	−0.0133 (10)
N2	0.0519 (11)	0.0357 (11)	0.0519 (13)	−0.0088 (9)	0.0105 (10)	−0.0082 (10)
C1	0.0501 (13)	0.0460 (14)	0.0292 (12)	0.0020 (10)	0.0201 (11)	−0.0068 (11)
C2	0.0744 (16)	0.0621 (17)	0.0389 (15)	0.0246 (13)	0.0150 (13)	−0.0026 (13)
C3	0.0810 (18)	0.0704 (19)	0.0333 (14)	0.0219 (15)	0.0131 (14)	−0.0020 (13)
C4	0.0655 (16)	0.0506 (15)	0.0399 (14)	0.0085 (12)	0.0234 (13)	−0.0078 (12)
C5	0.0673 (15)	0.0460 (15)	0.0408 (14)	0.0065 (11)	0.0221 (12)	−0.0012 (11)
C6	0.138 (3)	0.148 (3)	0.0490 (18)	0.071 (2)	0.0013 (19)	0.011 (2)
C7	0.177 (3)	0.073 (2)	0.073 (2)	−0.011 (2)	0.064 (2)	0.0078 (17)
C8	0.133 (3)	0.116 (3)	0.0400 (17)	0.027 (2)	0.0262 (18)	−0.0164 (17)
C9	0.0589 (16)	0.129 (3)	0.070 (2)	0.0144 (16)	0.0262 (15)	−0.0187 (18)
C10	0.151 (3)	0.0592 (19)	0.084 (2)	0.0199 (18)	0.055 (2)	−0.0183 (17)
C11	0.0412 (13)	0.0331 (12)	0.0317 (13)	−0.0006 (10)	0.0134 (11)	−0.0025 (10)
C12	0.0337 (11)	0.0313 (12)	0.0302 (11)	−0.0002 (9)	0.0134 (10)	−0.0018 (9)
C13	0.0404 (12)	0.0323 (13)	0.0298 (12)	0.0000 (10)	0.0120 (10)	−0.0014 (10)
C14	0.0373 (12)	0.0326 (13)	0.0699 (17)	−0.0081 (10)	0.0187 (12)	−0.0096 (12)
C15	0.0597 (15)	0.0466 (15)	0.0571 (17)	−0.0088 (12)	0.0094 (13)	0.0107 (13)
C16	0.0625 (16)	0.0527 (16)	0.0538 (17)	−0.0094 (12)	0.0017 (14)	0.0034 (14)
C17	0.0561 (14)	0.0350 (14)	0.0571 (16)	−0.0046 (11)	0.0236 (13)	0.0001 (12)
C18	0.0587 (14)	0.0410 (14)	0.0419 (14)	−0.0037 (11)	0.0201 (12)	−0.0067 (11)
C19	0.0642 (19)	0.073 (2)	0.134 (3)	−0.0026 (15)	−0.0278 (19)	0.024 (2)
C20	0.143 (3)	0.113 (3)	0.0535 (19)	−0.037 (2)	0.018 (2)	−0.0120 (19)
C21	0.0805 (18)	0.0462 (16)	0.088 (2)	−0.0164 (13)	0.0086 (16)	−0.0188 (15)
C22	0.105 (2)	0.0506 (17)	0.081 (2)	−0.0068 (15)	0.0141 (18)	0.0184 (16)
C23	0.0838 (19)	0.073 (2)	0.123 (3)	−0.0210 (16)	0.0640 (19)	−0.0103 (18)
C24	0.0392 (11)	0.0368 (13)	0.0393 (13)	−0.0024 (9)	0.0155 (10)	−0.0049 (10)
C25	0.0612 (15)	0.0411 (15)	0.0506 (15)	0.0058 (11)	0.0167 (13)	0.0033 (12)
C26	0.0808 (18)	0.0451 (16)	0.083 (2)	0.0121 (13)	0.0396 (16)	0.0094 (15)
C27	0.122 (3)	0.071 (2)	0.131 (3)	0.0336 (19)	0.030 (2)	0.043 (2)
C28	0.0362 (11)	0.0399 (13)	0.0342 (12)	0.0005 (9)	0.0121 (10)	0.0010 (10)
C29	0.0332 (11)	0.0382 (13)	0.0267 (11)	0.0016 (10)	0.0083 (9)	0.0044 (10)
C30	0.0391 (11)	0.0403 (13)	0.0262 (12)	−0.0011 (10)	0.0082 (10)	−0.0013 (10)
C31	0.0330 (11)	0.0453 (13)	0.0291 (12)	−0.0013 (10)	0.0081 (10)	0.0010 (11)
C32	0.0393 (12)	0.0508 (14)	0.0243 (12)	0.0034 (10)	0.0117 (10)	−0.0002 (11)
C33	0.0350 (11)	0.0375 (13)	0.0293 (12)	−0.0001 (10)	0.0056 (10)	0.0013 (10)
C34	0.0357 (11)	0.0409 (13)	0.0321 (12)	−0.0027 (10)	0.0088 (10)	0.0055 (11)
C35	0.0383 (12)	0.0516 (14)	0.0360 (13)	−0.0075 (10)	0.0135 (10)	−0.0006 (11)
C36	0.0630 (15)	0.0611 (17)	0.0757 (19)	−0.0231 (13)	0.0384 (14)	−0.0153 (14)
C37	0.0814 (18)	0.081 (2)	0.0544 (17)	−0.0234 (15)	0.0185 (15)	0.0149 (15)
C38	0.0458 (14)	0.0762 (18)	0.0735 (18)	−0.0095 (13)	0.0249 (13)	−0.0129 (15)
C39	0.0428 (12)	0.0423 (14)	0.0402 (13)	−0.0002 (11)	0.0079 (11)	−0.0070 (11)
C40	0.0672 (16)	0.0757 (19)	0.0423 (15)	−0.0060 (14)	0.0021 (13)	−0.0114 (14)
C41	0.0646 (16)	0.0589 (17)	0.083 (2)	0.0072 (13)	0.0141 (15)	−0.0181 (15)
C42	0.0752 (17)	0.0543 (16)	0.0656 (18)	−0.0193 (13)	0.0242 (15)	−0.0134 (14)

### Geometric parameters (Å, °)

**Table:**

O1—C11	1.191 (2)	C20—H20A	0.9600
O2—C11	1.333 (2)	C20—H20B	0.9600
O2—C1	1.463 (2)	C20—H20C	0.9600
O3—C13	1.328 (2)	C21—H21A	0.9600
O3—C14	1.458 (2)	C21—H21B	0.9600
O4—C13	1.195 (2)	C21—H21C	0.9600
O5—C32	1.389 (2)	C22—H22A	0.9600
O5—H5	0.8200	C22—H22B	0.9600
N1—C8	1.470 (3)	C22—H22C	0.9600
N1—C4	1.475 (3)	C23—H23A	0.9600
N1—C3	1.481 (3)	C23—H23B	0.9600
N2—C16	1.464 (3)	C23—H23C	0.9600
N2—C17	1.471 (3)	C24—C25	1.513 (3)
N2—C21	1.473 (3)	C24—H24A	0.9700
C1—C5	1.482 (3)	C24—H24B	0.9700
C1—C2	1.492 (3)	C25—C26	1.505 (3)
C1—H1	0.9800	C25—H25A	0.9700
C2—C3	1.535 (3)	C25—H25B	0.9700
C2—H2A	0.9700	C26—C27	1.508 (4)
C2—H2B	0.9700	C26—H26A	0.9700
C3—C6	1.524 (4)	C26—H26B	0.9700
C3—C7	1.543 (4)	C27—H27A	0.9600
C4—C5	1.526 (3)	C27—H27B	0.9600
C4—C10	1.527 (3)	C27—H27C	0.9600
C4—C9	1.529 (3)	C28—C29	1.516 (3)
C5—H5A	0.9700	C28—H28A	0.9700
C5—H5B	0.9700	C28—H28B	0.9700
C6—H6A	0.9600	C29—C34	1.375 (3)
C6—H6B	0.9600	C29—C30	1.380 (3)
C6—H6C	0.9600	C30—C31	1.394 (3)
C7—H7A	0.9600	C30—H30	0.9300
C7—H7B	0.9600	C31—C32	1.393 (3)
C7—H7C	0.9600	C31—C35	1.534 (3)
C8—H8A	0.9600	C32—C33	1.398 (3)
C8—H8B	0.9600	C33—C34	1.386 (3)
C8—H8C	0.9600	C33—C39	1.532 (3)
C9—H9A	0.9600	C34—H34	0.9300
C9—H9B	0.9600	C35—C36	1.526 (3)
C9—H9C	0.9600	C35—C37	1.532 (3)
C10—H10A	0.9600	C35—C38	1.533 (3)
C10—H10B	0.9600	C36—H36A	0.9600
C10—H10C	0.9600	C36—H36B	0.9600
C11—C12	1.525 (3)	C36—H36C	0.9600
C12—C13	1.524 (3)	C37—H37A	0.9600
C12—C24	1.535 (3)	C37—H37B	0.9600
C12—C28	1.551 (3)	C37—H37C	0.9600
C14—C18	1.481 (3)	C38—H38A	0.9600
C14—C15	1.503 (3)	C38—H38B	0.9600
C14—H14	0.9800	C38—H38C	0.9600
C15—C16	1.533 (3)	C39—C42	1.529 (3)
C15—H15A	0.9700	C39—C40	1.531 (3)
C15—H15B	0.9700	C39—C41	1.539 (3)
C16—C19	1.538 (4)	C40—H40A	0.9600
C16—C20	1.544 (4)	C40—H40B	0.9600
C17—C22	1.523 (3)	C40—H40C	0.9600
C17—C18	1.528 (3)	C41—H41A	0.9600
C17—C23	1.545 (3)	C41—H41B	0.9600
C18—H18A	0.9700	C41—H41C	0.9600
C18—H18B	0.9700	C42—H42A	0.9600
C19—H19A	0.9600	C42—H42B	0.9600
C19—H19B	0.9600	C42—H42C	0.9600
C19—H19C	0.9600		
			
C11—O2—C1	118.27 (15)	H20A—C20—H20B	109.5
C13—O3—C14	120.73 (15)	C16—C20—H20C	109.5
C32—O5—H5	109.5	H20A—C20—H20C	109.5
C8—N1—C4	112.82 (19)	H20B—C20—H20C	109.5
C8—N1—C3	112.4 (2)	N2—C21—H21A	109.5
C4—N1—C3	119.17 (17)	N2—C21—H21B	109.5
C16—N2—C17	118.96 (18)	H21A—C21—H21B	109.5
C16—N2—C21	112.47 (18)	N2—C21—H21C	109.5
C17—N2—C21	113.32 (18)	H21A—C21—H21C	109.5
O2—C1—C5	110.61 (17)	H21B—C21—H21C	109.5
O2—C1—C2	105.78 (16)	C17—C22—H22A	109.5
C5—C1—C2	109.58 (18)	C17—C22—H22B	109.5
O2—C1—H1	110.3	H22A—C22—H22B	109.5
C5—C1—H1	110.3	C17—C22—H22C	109.5
C2—C1—H1	110.3	H22A—C22—H22C	109.5
C1—C2—C3	113.23 (18)	H22B—C22—H22C	109.5
C1—C2—H2A	108.9	C17—C23—H23A	109.5
C3—C2—H2A	108.9	C17—C23—H23B	109.5
C1—C2—H2B	108.9	H23A—C23—H23B	109.5
C3—C2—H2B	108.9	C17—C23—H23C	109.5
H2A—C2—H2B	107.7	H23A—C23—H23C	109.5
N1—C3—C6	108.6 (2)	H23B—C23—H23C	109.5
N1—C3—C2	107.9 (2)	C25—C24—C12	118.61 (17)
C6—C3—C2	105.8 (2)	C25—C24—H24A	107.7
N1—C3—C7	115.1 (2)	C12—C24—H24A	107.7
C6—C3—C7	108.7 (2)	C25—C24—H24B	107.7
C2—C3—C7	110.3 (2)	C12—C24—H24B	107.7
N1—C4—C5	108.04 (18)	H24A—C24—H24B	107.1
N1—C4—C10	107.3 (2)	C26—C25—C24	112.54 (19)
C5—C4—C10	106.2 (2)	C26—C25—H25A	109.1
N1—C4—C9	115.8 (2)	C24—C25—H25A	109.1
C5—C4—C9	110.3 (2)	C26—C25—H25B	109.1
C10—C4—C9	108.7 (2)	C24—C25—H25B	109.1
C1—C5—C4	113.70 (18)	H25A—C25—H25B	107.8
C1—C5—H5A	108.8	C25—C26—C27	113.3 (2)
C4—C5—H5A	108.8	C25—C26—H26A	108.9
C1—C5—H5B	108.8	C27—C26—H26A	108.9
C4—C5—H5B	108.8	C25—C26—H26B	108.9
H5A—C5—H5B	107.7	C27—C26—H26B	108.9
C3—C6—H6A	109.5	H26A—C26—H26B	107.7
C3—C6—H6B	109.5	C26—C27—H27A	109.5
H6A—C6—H6B	109.5	C26—C27—H27B	109.5
C3—C6—H6C	109.5	H27A—C27—H27B	109.5
H6A—C6—H6C	109.5	C26—C27—H27C	109.5
H6B—C6—H6C	109.5	H27A—C27—H27C	109.5
C3—C7—H7A	109.5	H27B—C27—H27C	109.5
C3—C7—H7B	109.5	C29—C28—C12	115.05 (15)
H7A—C7—H7B	109.5	C29—C28—H28A	108.5
C3—C7—H7C	109.5	C12—C28—H28A	108.5
H7A—C7—H7C	109.5	C29—C28—H28B	108.5
H7B—C7—H7C	109.5	C12—C28—H28B	108.5
N1—C8—H8A	109.5	H28A—C28—H28B	107.5
N1—C8—H8B	109.5	C34—C29—C30	117.61 (18)
H8A—C8—H8B	109.5	C34—C29—C28	121.44 (18)
N1—C8—H8C	109.5	C30—C29—C28	120.95 (19)
H8A—C8—H8C	109.5	C29—C30—C31	123.05 (19)
H8B—C8—H8C	109.5	C29—C30—H30	118.5
C4—C9—H9A	109.5	C31—C30—H30	118.5
C4—C9—H9B	109.5	C32—C31—C30	116.44 (18)
H9A—C9—H9B	109.5	C32—C31—C35	122.89 (18)
C4—C9—H9C	109.5	C30—C31—C35	120.67 (19)
H9A—C9—H9C	109.5	O5—C32—C31	120.39 (18)
H9B—C9—H9C	109.5	O5—C32—C33	116.58 (19)
C4—C10—H10A	109.5	C31—C32—C33	122.98 (18)
C4—C10—H10B	109.5	C34—C33—C32	116.60 (19)
H10A—C10—H10B	109.5	C34—C33—C39	121.39 (18)
C4—C10—H10C	109.5	C32—C33—C39	121.99 (18)
H10A—C10—H10C	109.5	C29—C34—C33	123.26 (19)
H10B—C10—H10C	109.5	C29—C34—H34	118.4
O1—C11—O2	123.79 (19)	C33—C34—H34	118.4
O1—C11—C12	124.9 (2)	C36—C35—C37	106.6 (2)
O2—C11—C12	111.18 (17)	C36—C35—C38	105.98 (18)
C13—C12—C11	109.16 (16)	C37—C35—C38	110.76 (19)
C13—C12—C24	112.53 (15)	C36—C35—C31	111.67 (17)
C11—C12—C24	108.59 (15)	C37—C35—C31	111.30 (17)
C13—C12—C28	109.08 (15)	C38—C35—C31	110.38 (18)
C11—C12—C28	107.70 (15)	C35—C36—H36A	109.5
C24—C12—C28	109.66 (15)	C35—C36—H36B	109.5
O4—C13—O3	124.26 (18)	H36A—C36—H36B	109.5
O4—C13—C12	126.58 (19)	C35—C36—H36C	109.5
O3—C13—C12	109.12 (17)	H36A—C36—H36C	109.5
O3—C14—C18	106.68 (17)	H36B—C36—H36C	109.5
O3—C14—C15	107.08 (17)	C35—C37—H37A	109.5
C18—C14—C15	108.72 (18)	C35—C37—H37B	109.5
O3—C14—H14	111.4	H37A—C37—H37B	109.5
C18—C14—H14	111.4	C35—C37—H37C	109.5
C15—C14—H14	111.4	H37A—C37—H37C	109.5
C14—C15—C16	114.51 (19)	H37B—C37—H37C	109.5
C14—C15—H15A	108.6	C35—C38—H38A	109.5
C16—C15—H15A	108.6	C35—C38—H38B	109.5
C14—C15—H15B	108.6	H38A—C38—H38B	109.5
C16—C15—H15B	108.6	C35—C38—H38C	109.5
H15A—C15—H15B	107.6	H38A—C38—H38C	109.5
N2—C16—C15	108.42 (19)	H38B—C38—H38C	109.5
N2—C16—C19	115.5 (2)	C42—C39—C40	106.97 (18)
C15—C16—C19	110.4 (2)	C42—C39—C33	111.77 (18)
N2—C16—C20	108.4 (2)	C40—C39—C33	110.21 (18)
C15—C16—C20	105.6 (2)	C42—C39—C41	106.84 (19)
C19—C16—C20	108.0 (3)	C40—C39—C41	109.55 (19)
N2—C17—C22	108.62 (19)	C33—C39—C41	111.35 (17)
N2—C17—C18	108.33 (17)	C39—C40—H40A	109.5
C22—C17—C18	106.05 (19)	C39—C40—H40B	109.5
N2—C17—C23	115.6 (2)	H40A—C40—H40B	109.5
C22—C17—C23	107.6 (2)	C39—C40—H40C	109.5
C18—C17—C23	110.16 (19)	H40A—C40—H40C	109.5
C14—C18—C17	113.36 (18)	H40B—C40—H40C	109.5
C14—C18—H18A	108.9	C39—C41—H41A	109.5
C17—C18—H18A	108.9	C39—C41—H41B	109.5
C14—C18—H18B	108.9	H41A—C41—H41B	109.5
C17—C18—H18B	108.9	C39—C41—H41C	109.5
H18A—C18—H18B	107.7	H41A—C41—H41C	109.5
C16—C19—H19A	109.5	H41B—C41—H41C	109.5
C16—C19—H19B	109.5	C39—C42—H42A	109.5
H19A—C19—H19B	109.5	C39—C42—H42B	109.5
C16—C19—H19C	109.5	H42A—C42—H42B	109.5
H19A—C19—H19C	109.5	C39—C42—H42C	109.5
H19B—C19—H19C	109.5	H42A—C42—H42C	109.5
C16—C20—H20A	109.5	H42B—C42—H42C	109.5
C16—C20—H20B	109.5		
			
C11—O2—C1—C5	−86.2 (2)	C14—C15—C16—C19	−77.4 (3)
C11—O2—C1—C2	155.23 (18)	C14—C15—C16—C20	166.1 (2)
O2—C1—C2—C3	176.37 (19)	C16—N2—C17—C22	166.47 (19)
C5—C1—C2—C3	57.1 (3)	C21—N2—C17—C22	−57.9 (2)
C8—N1—C3—C6	−59.7 (3)	C16—N2—C17—C18	51.7 (2)
C4—N1—C3—C6	165.0 (2)	C21—N2—C17—C18	−172.70 (17)
C8—N1—C3—C2	−173.9 (2)	C16—N2—C17—C23	−72.5 (2)
C4—N1—C3—C2	50.7 (3)	C21—N2—C17—C23	63.1 (2)
C8—N1—C3—C7	62.4 (3)	O3—C14—C18—C17	172.24 (17)
C4—N1—C3—C7	−72.9 (3)	C15—C14—C18—C17	57.1 (2)
C1—C2—C3—N1	−52.0 (3)	N2—C17—C18—C14	−54.1 (2)
C1—C2—C3—C6	−168.0 (2)	C22—C17—C18—C14	−170.6 (2)
C1—C2—C3—C7	74.5 (3)	C23—C17—C18—C14	73.2 (3)
C8—N1—C4—C5	174.1 (2)	C13—C12—C24—C25	60.3 (2)
C3—N1—C4—C5	−50.7 (3)	C11—C12—C24—C25	−60.6 (2)
C8—N1—C4—C10	60.0 (3)	C28—C12—C24—C25	−178.05 (16)
C3—N1—C4—C10	−164.8 (2)	C12—C24—C25—C26	175.83 (18)
C8—N1—C4—C9	−61.7 (3)	C24—C25—C26—C27	−177.1 (2)
C3—N1—C4—C9	73.5 (3)	C13—C12—C28—C29	62.9 (2)
O2—C1—C5—C4	−173.54 (18)	C11—C12—C28—C29	−178.77 (17)
C2—C1—C5—C4	−57.3 (3)	C24—C12—C28—C29	−60.8 (2)
N1—C4—C5—C1	52.2 (3)	C12—C28—C29—C34	99.7 (2)
C10—C4—C5—C1	167.1 (2)	C12—C28—C29—C30	−81.4 (2)
C9—C4—C5—C1	−75.3 (3)	C34—C29—C30—C31	−1.3 (3)
C1—O2—C11—O1	1.2 (3)	C28—C29—C30—C31	179.76 (18)
C1—O2—C11—C12	−175.33 (15)	C29—C30—C31—C32	−0.6 (3)
O1—C11—C12—C13	143.1 (2)	C29—C30—C31—C35	−179.78 (18)
O2—C11—C12—C13	−40.4 (2)	C30—C31—C32—O5	179.72 (18)
O1—C11—C12—C24	−93.9 (2)	C35—C31—C32—O5	−1.1 (3)
O2—C11—C12—C24	82.60 (19)	C30—C31—C32—C33	2.4 (3)
O1—C11—C12—C28	24.8 (3)	C35—C31—C32—C33	−178.40 (18)
O2—C11—C12—C28	−158.72 (16)	O5—C32—C33—C34	−179.63 (17)
C14—O3—C13—O4	−8.6 (3)	C31—C32—C33—C34	−2.2 (3)
C14—O3—C13—C12	173.73 (17)	O5—C32—C33—C39	−0.9 (3)
C11—C12—C13—O4	131.1 (2)	C31—C32—C33—C39	176.51 (18)
C24—C12—C13—O4	10.5 (3)	C30—C29—C34—C33	1.5 (3)
C28—C12—C13—O4	−111.4 (2)	C28—C29—C34—C33	−179.55 (18)
C11—C12—C13—O3	−51.2 (2)	C32—C33—C34—C29	0.2 (3)
C24—C12—C13—O3	−171.84 (16)	C39—C33—C34—C29	−178.58 (18)
C28—C12—C13—O3	66.2 (2)	C32—C31—C35—C36	177.27 (19)
C13—O3—C14—C18	135.29 (19)	C30—C31—C35—C36	−3.6 (3)
C13—O3—C14—C15	−108.4 (2)	C32—C31—C35—C37	58.3 (3)
O3—C14—C15—C16	−170.24 (18)	C30—C31—C35—C37	−122.5 (2)
C18—C14—C15—C16	−55.3 (3)	C32—C31—C35—C38	−65.1 (3)
C17—N2—C16—C15	−49.6 (3)	C30—C31—C35—C38	114.0 (2)
C21—N2—C16—C15	174.4 (2)	C34—C33—C39—C42	−2.7 (3)
C17—N2—C16—C19	74.9 (3)	C32—C33—C39—C42	178.66 (19)
C21—N2—C16—C19	−61.1 (3)	C34—C33—C39—C40	116.1 (2)
C17—N2—C16—C20	−163.8 (2)	C32—C33—C39—C40	−62.5 (2)
C21—N2—C16—C20	60.3 (3)	C34—C33—C39—C41	−122.1 (2)
C14—C15—C16—N2	50.1 (3)	C32—C33—C39—C41	59.3 (3)

### Hydrogen-bond geometry (Å, °)

**Table:**

*D*—H···*A*	*D*—H	H···*A*	*D*···*A*	*D*—H···*A*
O5—H5···O4^i^	0.82	2.58	3.200 (3)	134

Symmetry codes: (i) −*x*+1, −*y*, −*z*.
